# Imaging of the adult male urethra, penile prostheses and artificial urinary sphincters

**DOI:** 10.1007/s00261-019-02356-x

**Published:** 2019-12-13

**Authors:** Subramaniyan Ramanathan, Vineetha Raghu, Parvati Ramchandani

**Affiliations:** 1 Department of Radiology, Al-Wakra Hospital, Hamad Medical Corporation, PO Box: 82228, Doha, Qatar; 2 Department of Radiology, Weil Cornell Medical College, Doha, Qatar; 3 Department of Radiology, Columbia Asia Referral Hospital, Yeshwanthpur, India; 4 Department of Radiology and Surgery, Perelman School of Medicine at the University of Pennsylvania, Philadelphia, PA 19104, USA

**Keywords:** Urethrography, Male urethra, Penile prostheses, Artifical urinary sphincters, MRI, Urethral stricture

## Abstract

To discuss the imaging appearances of various pathologies affecting adult male urethra and to review the role of imaging in the assessment of artificial urinary sphincters and penile prostheses. Diagnosis of common male urethral diseases heavily depends on two conventional fluoroscopic techniques namely retrograde urethrography and voiding cystourethrography. These are useful in evaluating common urethral diseases like traumatic injury, infections, and strictures. Cross-sectional imaging can be useful in evaluating periurethral pathologies. Artificial urinary sphincters, slings, and periurethral bulking agents are used in the management of urinary incontinence and imaging can be utilized to detect complications in these devices. Cross-sectional imaging especially MRI plays a significant role in evaluating the different types of penile prostheses and their malfunctioning.

## Introduction

Evaluation of the urethra is indicated in trauma, inflammatory pathologies, strictures due to any cause, and for post-operative assessment. Retrograde urethrography (RUG) and voiding cystourethrography (VCUG) are the two common fluoroscopic contrast techniques used currently to assess the anterior and posterior urethra, respectively, for the many indications mentioned above. Ultrasound (US), computed tomography (CT), and magnetic resonance imaging (MRI) are necessary for the evaluation of periurethral structures. MRI is the gold standard for evaluation of urethral diverticula and urethral tumors.

Urinary incontinence and erectile dysfunction are common elderly male urological problems. Various treatment options available include artificial sphincters, periurethral bulking agents, and penile implants. Imaging plays a key role in the evaluation of these artificial devices specifically to detect the complications. In this review, we discuss the various imaging modalities useful in the evaluation of male urethra, with a note on artificial urinary sphincters and penile prostheses. Evaluation of other penile conditions is discussed elsewhere in this issue.

## Anatomy of the male urethra

The male urethra measures 17 to 20 cm in length and is divided into anterior and posterior segments. The anterior urethra is further subdivided into the penile and bulbar parts, and the posterior urethra into the membranous and prostatic segments. The prostatic urethra courses through the prostate gland and has two specific landmarks. The verumontanum is an ovoid mound of tissue on the posterior wall, housing the utricle and ejaculatory duct openings. The membranous urethra is the shortest and least distensible segment of the urethra, narrowest only second to the meatus. The bulbo-membranous junction is located at the inferior margin of the urogenital diaphragm, and corresponds to the “cone” seen on fluoroscopic contrast studies. The penile urethra measures about 20 cm in length, and ends at the external urethral meatus. The distal end of the penile urethra located in the glans penis is dilated to form the ‘fossa navicularis’ and is approximately 1.5 cm long. Surrounding the urethra are lubricating glands: the pea-sized paired Cowper glands located within the urogenital diaphragm which drain into the bulbar urethra, and the periurethral glands of Littre, most abundant in the dorsal penile urethra and the bulbous urethral sump [1, 2].

### Urethral sphincters

The internal urethral sphincter is a cuff of smooth muscle extending from the bladder neck to the prostatic urethra, above the verumontanum, and is essential for passive urinary continence.

The external urethral sphincter is divided into intrinsic and extrinsic segments. The intrinsic external sphincter is smooth muscle along the distal prostatic and membranous urethra, also involved in passive continence, while the extrinsic external sphincter is a paraurethral voluntary muscle which surrounds the membranous urethra and is responsible for active continence [1, 3].

## Technique of retrograde urethrography (RUG)

The RUG remains an invaluable technique for assessing the anterior urethra. A scout film (KUB radiograph) is initially performed to look for bony abnormalities or calcification and should ideally extend to at least a few centimeters below the pubic symphysis. A 14 to 18F Foley catheter is inserted through the external meatus with aseptic technique. A smaller (8F or 10F) can be used in patients with meatal stenosis to avoid iatrogenic trauma. The catheter must be flushed before insertion to get rid of air bubbles which may be mistaken for filling defects within the urethra. The balloon of the catheter is placed in the fossa navicularis and inflated with 1.5 ml fluid, either contrast or saline; air should not be used to inflate the balloon as it is more compressible than fluid and inadvertent balloon withdrawal can occur during the study. It is best not to lubricate the catheter prior to placement to prevent slippage of the catheter from the glans. The patient is positioned supine with a 45° oblique tilt, and dependent hip flexed. The penis is placed sideways over the thigh with moderate traction; oblique positioning of the patient and penile traction are necessary to straighten the urethra, particularly at the penoscrotal junction. 20–30 ml of iodinated contrast is injected under fluoroscopic guidance.

In young patients with high-resting muscular tone in the external sphincter, contrast is unlikely to flow retrograde past the bulbo-membranous urethral junction. In older patients, slow and gentle injection may often result in some contrast flowing past the external sphincter to opacify the posterior urethra, but optimal evaluation of the posterior urethra requires a VCUG [2, 4].

## Technique of voiding cystourethrography (VCUG)

VCUG is a method to delineate the anatomy and pathology of the posterior urethra. In this technique, the bladder is filled with contrast through a Foley catheter, suprapubic catheter or performed at the end of excretory urogram (the latter produces less dense contrast and may not result in optimal visualization of the urethra). The volume of contrast depends on the individual’s bladder capacity, which can vary from 300 to 800 ml, but it is necessary to fill until the patient reports a strong urge to void. Voiding is performed under fluoroscopic control in upright 45° oblique position or supine oblique position on a horizontal table in patients unable to stand [1, 4].

### Normal appearance

RUG provides excellent delineation of the urethra. The bulbar cone at the bulbo-membranous junction is important to identify, to assess proximal disease extent and plan urologic procedures. It lies about 1–1.5 cm distal to the inferior margin of the verumontanum and corresponds to an imaginary line at the level of inferior margins of obturator foramina [2, 5].

**Fig. 1:**
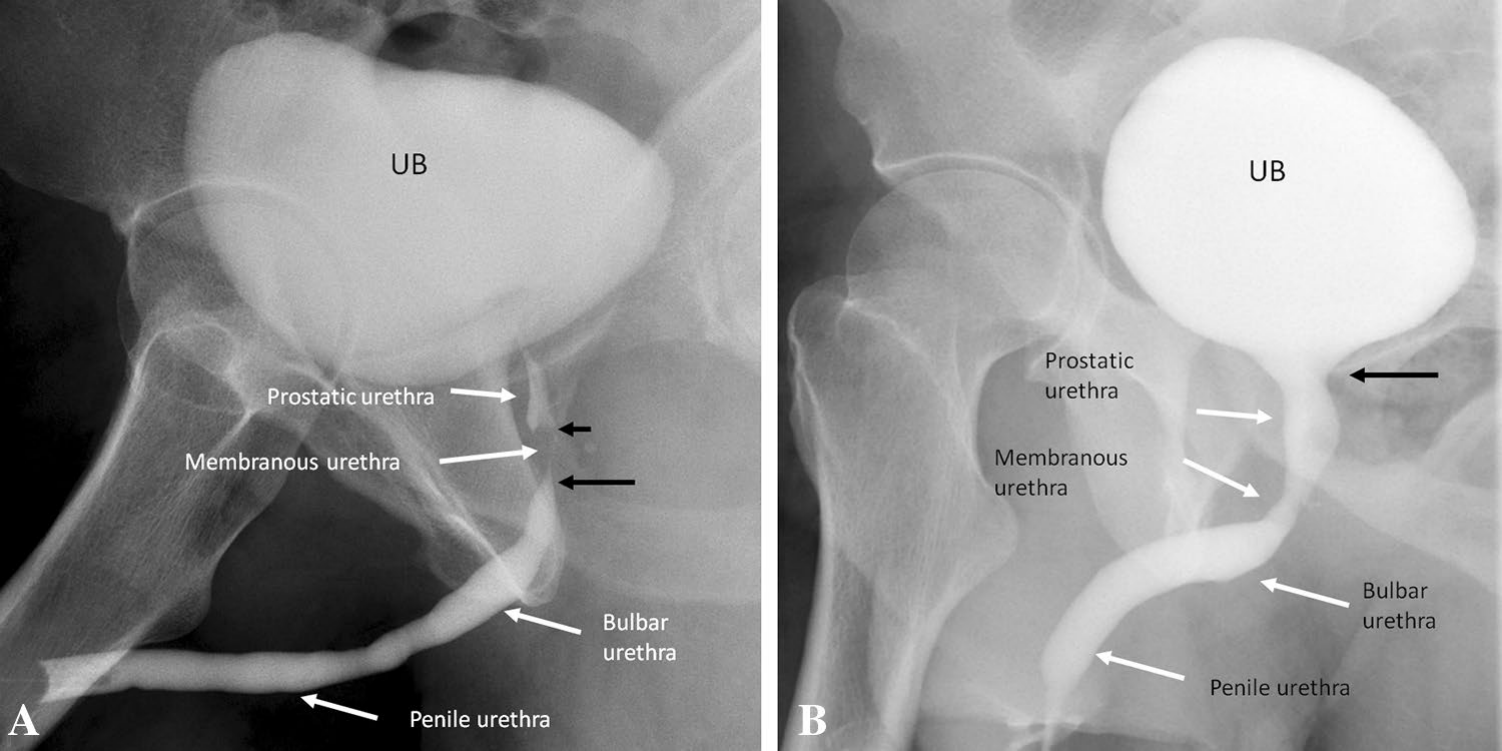
Normal urethral anatomy. **a** Retrograde urethrogram shows the anatomical landmarks. The verumontanum is seen as triangular structure in the posterior prostatic urethra (short black arrow). The bulbar cone (distal membranous urethra) is important to identify, to assess disease and plan urologic procedures (long black arrow). **b** Voiding cystourethrogram shows the normal urethral segments with wide bladder neck (black arrow) during voiding

A contrast jet from the bladder neck to the urinary bladder may be observed if contrast flows retrograde past the external sphincter. The verumontanum may be identified as a diamond-shaped mound in the posterior prostatic urethra if there is retrograde opacification of the posterior urethra (Fig. 1).

## Role of imaging in the evaluation of male urethra

Although conventional urethrographic techniques help in excellent evaluation of the luminal pathologies, cross-sectional modalities such as US, CT, and MRI are indicated in certain cases of periurethral abnormalities and enable comprehensive evaluation of the male urethra.

US with a high frequency linear probe (7.5 to 10 MHz) along with retrograde injection of normal saline known as sonourethrography helps in the evaluation of periurethral tissues, however, has a small field of view and is operator dependent [6]. Nonetheless, it has been found to be effective in the evaluation of anterior urethral strictures, their length and extent, as well as associated periurethral spongiofibrosis [7].

CT (polytrauma protocol) is often the first imaging modality in the evaluation of blunt abdominopelvic trauma. Radiologists focus on the pelvic fractures and bladder injury and urethral trauma is often overlooked as it is usually diagnosed on urethrography. CT may demonstrate periurethral hematoma, urinary leak above or below the urogenital diaphragm, displacement of the prostatic apex and pelvic hematomas in cases of posterior urethral injury which is unusual without pelvic fractures [8]. CT voiding urethrography has also been described with good results in patients with urethral trauma, stricture, and congenital abnormalities such as hypospadiasis [9].

MRI is the modality of choice for the demonstration of periurethral soft tissues, and being non-invasive with superior contrast resolution and multiplanar imaging capabilities, serves as a useful adjunctive tool in several congenital, inflammatory, and neoplastic pathologies. Intersex or complex genitourinary anomalies may need MRI assessment for the evaluation of pelvic and genitourinary anatomy and fistulous communications between pelvic organs. In urethritis, MRI may demonstrate periurethral abscesses and sinus tracts which are not assessed on conventional urethrographic techniques. MRI is also the investigation of choice for the characterization, local staging of urethral tumors and enables treatment planning [10].

**Fig. 2:**
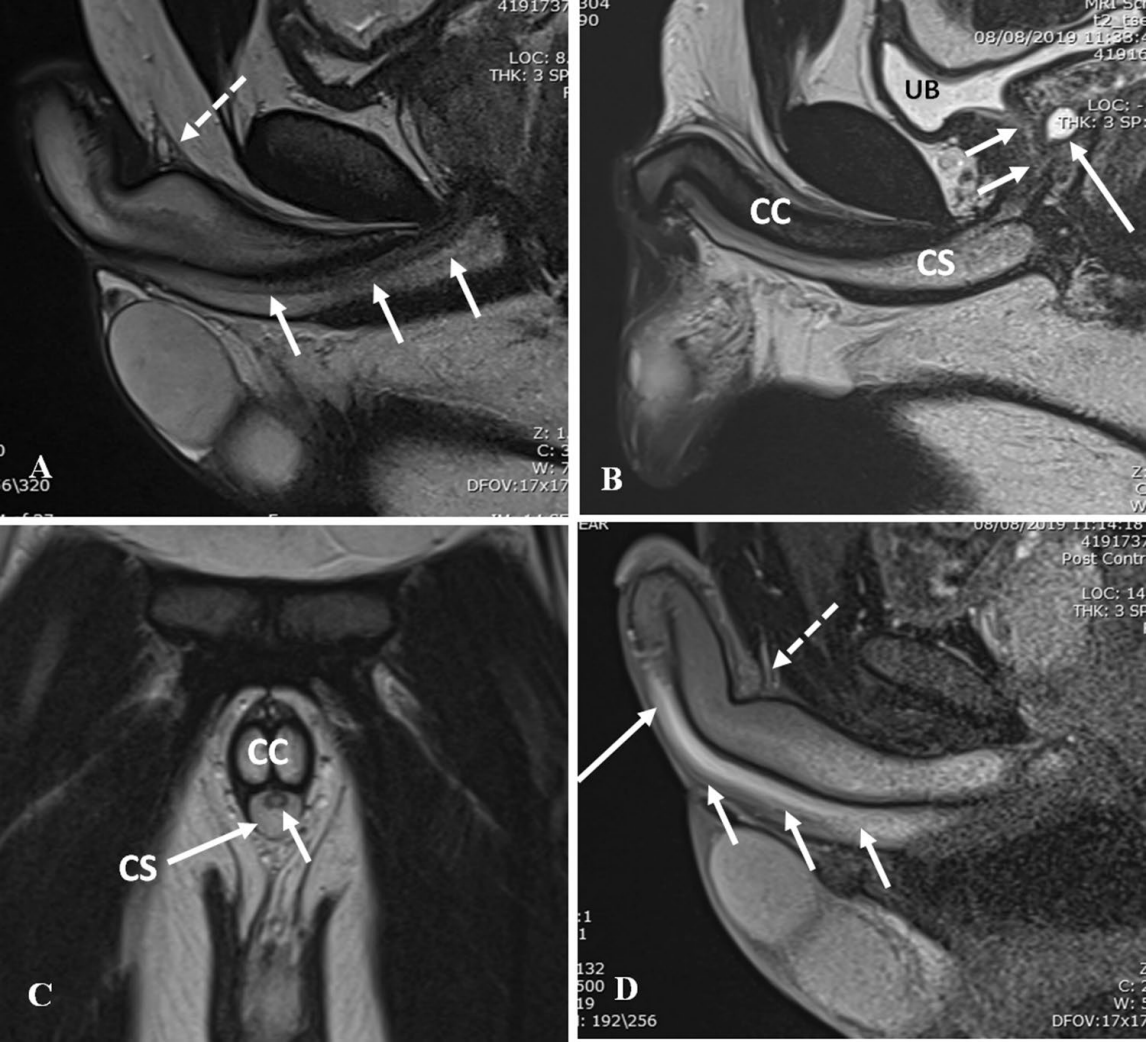
Normal urethral anatomy on MRI. **a** Sagittal T2-weighted MRI shows the bulbar and penile urethra (arrows). Suspensory ligament is shown inserting along the dorsal aspect of anterior urethra (dashed arrows) dividing the fixed and mobile segments of anterior urethra. **b** Sagittal T2-weighted MRI shows the prostatic urethra (short arrows) with incidental prostatic utricle cyst (long arrows). *CS* corpus spongiosum, *CC* corpus cavernosa, *UB* urinary bladder. **c** Coronal T2-weighted MRI shows the penile urethra (short arrow) lying within the corpus spongiosum (CS). *CC* corpus cavernosa. **d** Sagittal post-contrast T1-weighted MRI shows the enhancement along the bulbar urethra (short arrows) and penile urethra (long arrow). Suspensory ligament is shown inserting along the dorsal aspect of anterior urethra (dashed arrows)

T1- and T2-weighted images are used, along with intravenous gadolinium based contrast in certain cases, with images acquired in orthogonal (axial, sagittal, and coronal) planes for evaluation of the posterior urethra. Images may also be acquired obliquely along the course of the urethra, especially while evaluating the anterior urethra. The penis may be taped to the abdominal wall for better visualization. Thin sections of 3–5 mm with a small intersection gap of 1–2 mm provide images of adequate diagnostic value (Fig. 2) [11].

## Urethral trauma

The male urethra may be injured by blunt or penetrating trauma. Clinically, there may be blood at the meatus (approximately 50% of cases), and this should prompt a RUG prior to attempted urethral catheterization, in order to prevent exacerbation of an injury. Urethral injuries may lead to strictures, fistulae (usually due to iatrogenic injuries, often as a complication of radiation therapy or surgery), incontinence (if the external sphincter is involved), and impotence with severe posterior urethral injuries in association with pelvic fractures [5].

**Fig. 3:**
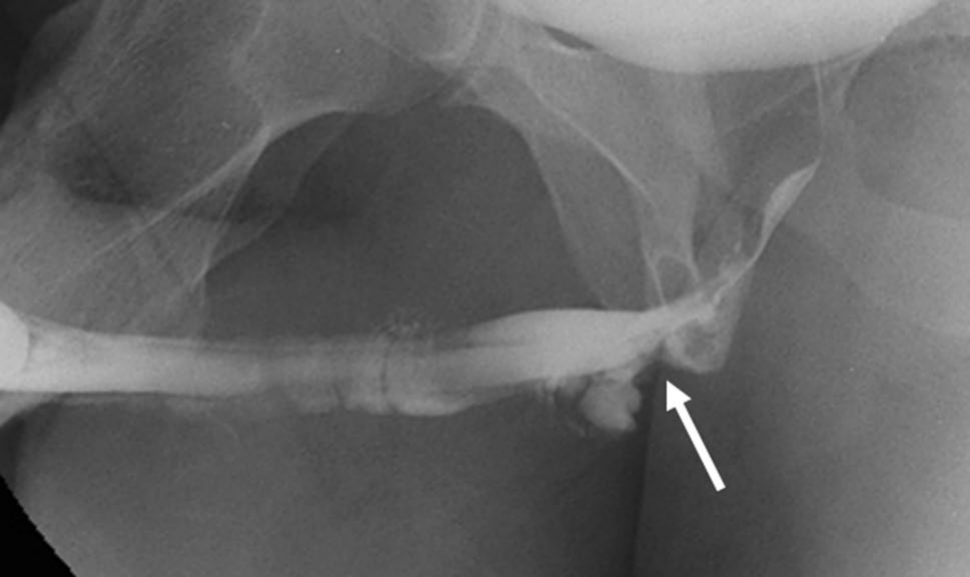
Retrograde urethrogram in a case of urethral trauma demonstrates near-complete disruption of the proximal bulbous urethra (white arrow) with venous intravasation

Blunt urethral injuries may further be classified as anterior urethral injury and posterior urethral injury. Anterior urethral injury is usually isolated and is a “straddle” type of pelvic injury where the bulbar urethra is compressed against the symphysis pubis by a hard object. A common mechanism is bicycle handlebar injuries, and playground equipment injuries in young children. Partial disruption of the anterior urethra is more common than a complete tear. It is usually not associated with bony injury, and may be complicated by stricture and impotence. RUG demonstrates disruption of the proximal bulbar urethra with venous opacification due to intravasation of contrast [12–14] (Fig. 3).

**Fig. 4:**
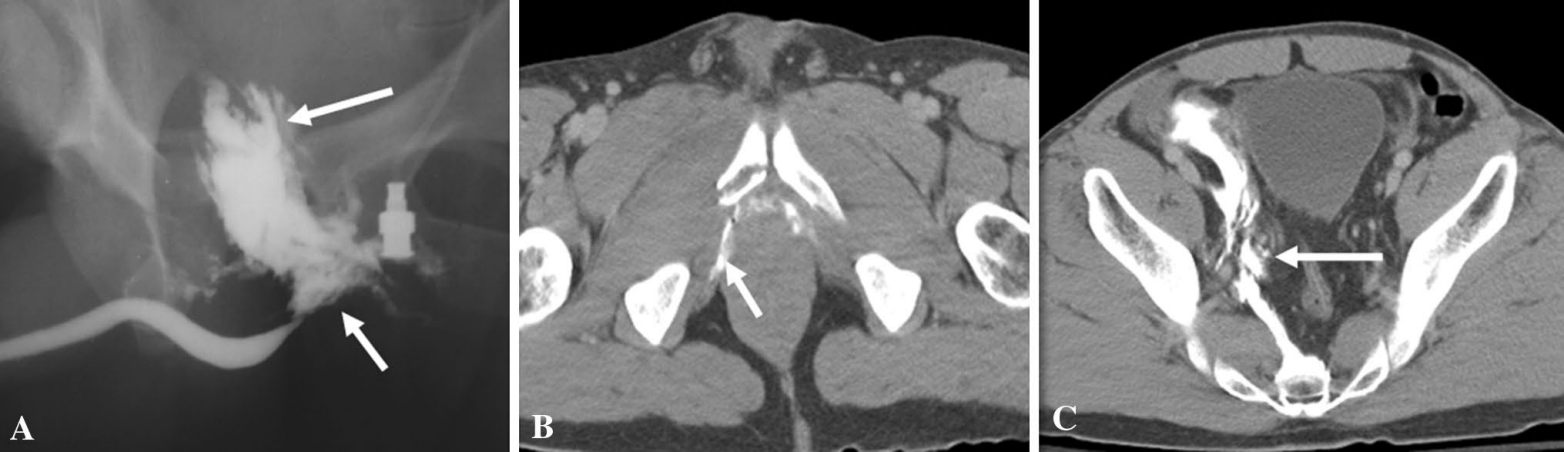
Colapinto–McCallum type III injury. **a** Retrograde urethrogram shows contrast extravasation from the posterior urethra extending into perineum (short arrow) and pelvis (long arrow). **b**, **c** Axial computed tomography of pelvis confirms the perineal (short arrow) and extraperitoneal pelvic extravasation (long arrow)

Posterior urethral injuries are associated with pelvic injuries and pelvic fractures (4–14%) due to motor vehicle collisions, motorcycle accidents, or crush injuries [12]. Lacerations of the urinary bladder are associated in 20%. The Colapinto–McCallum classification of posterior urethral injuries (Types I, II, and III) is described below [15] (Fig. 4).

Type II and Type III injuries result in dislocation of the bladder out of the pelvis and are associated with external sphincter damage and incontinence.

**Fig. 5:**
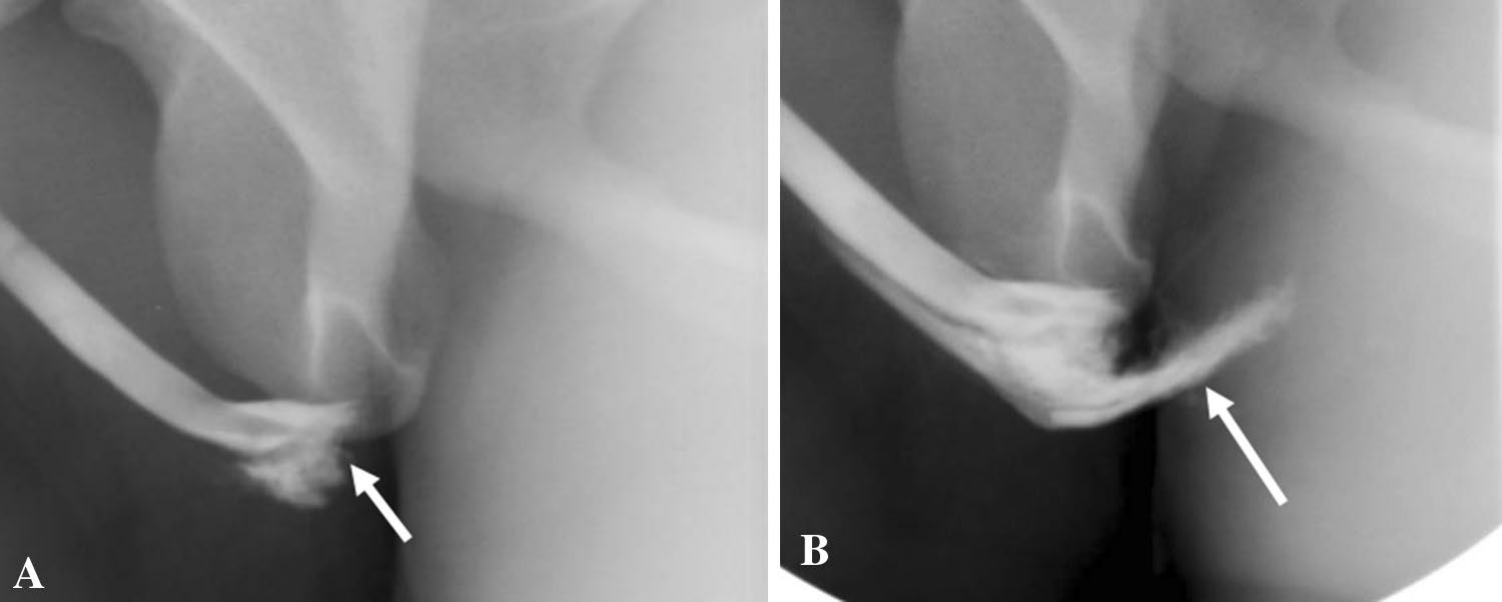
Type V straddle anterior urethral injury. **a**, **b** Retrograde urethrogram shows complete disruption of proximal bulbar urethra (short arrow) with extensive venous intravasation (long arrow)

Goldman et al. have described additional types IV and V, with the type V injury actually representing an anterior urethra injury (Fig. 5) [16].

**Fig. 6:**
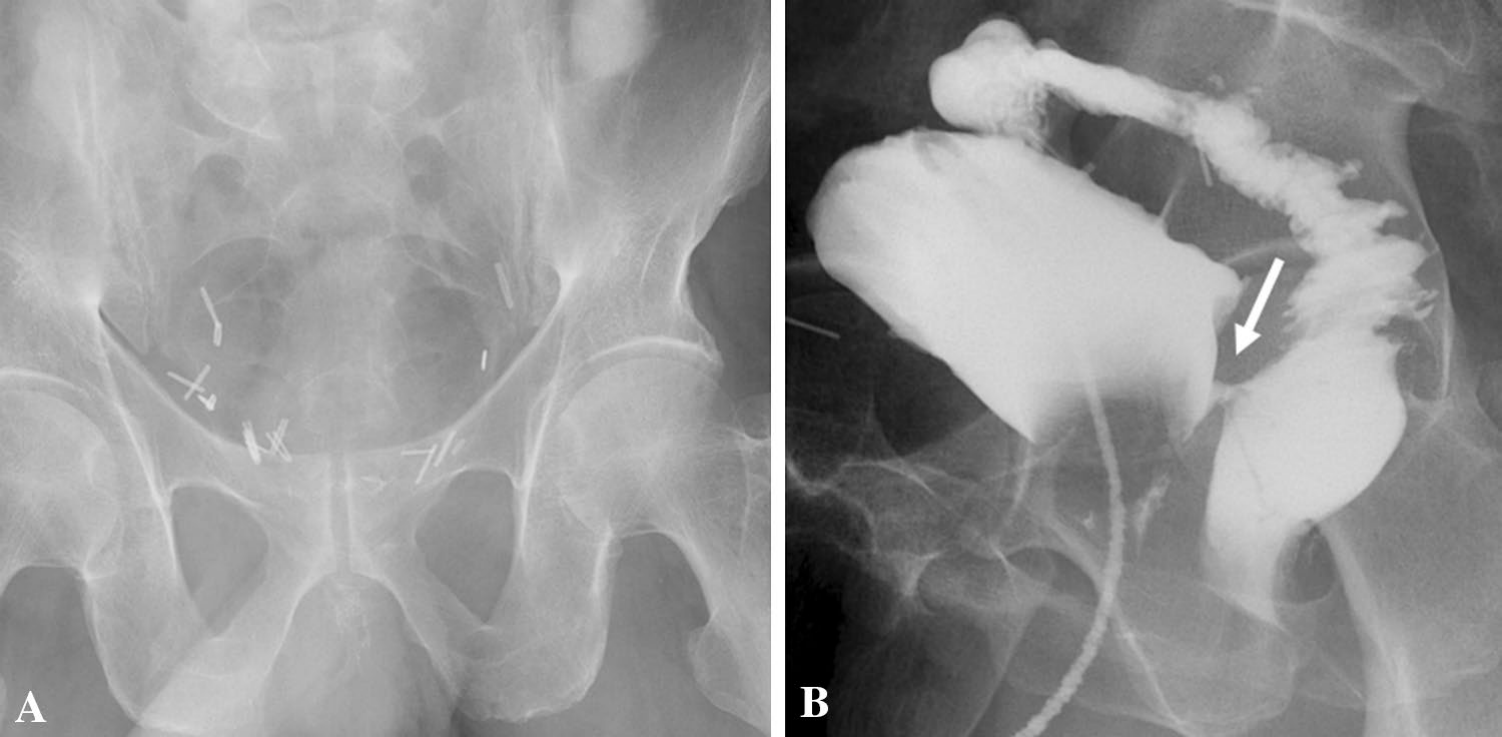
Iatrogenic posterior urethral injury. Plain radiograph (**a**) and voiding cystourethrogram (**b**) in an elderly gentleman with a prostato-rectal fistula (arrow), post-abdominoperineal resection and radical prostatectomy

Types IV and IVa urethral injuries are radiographically indistinguishable. [13, 17]. Iatrogenic posterior urethral injury usually requires a VCUG for diagnosis (Fig. 6) [14, 18].

**Fig. 7:**
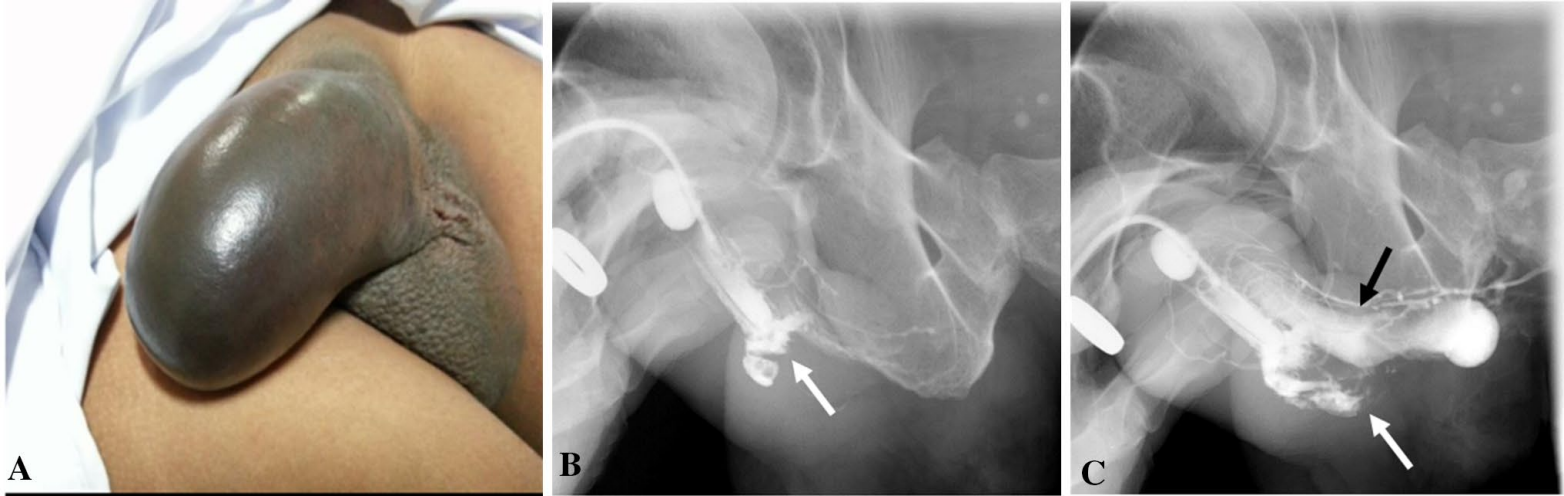
Penile fracture with urethral injury. **a** Clinical photograph shows the eggplant deformity in penile fracture. **b**, **c** Retrograde urethrogram shows bulbar urethral disruption with contrast extravasation (white arrow) and opacification of the corpora cavernosa (black arrow) indicating an urethrocavernous fistula

**Fig. 8:**
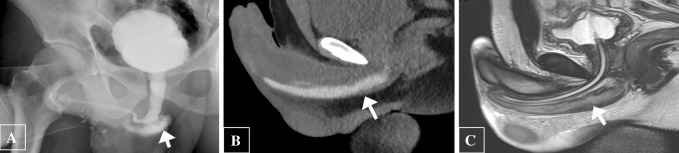
Urethral and corpus spongiosum injury in a 21-year-old gentleman with penile swelling and hematuria post intercourse. **a** Retrograde urethrogram depicts a urethral laceration with contrast leak into the corpus spongiosum (arrow). **b** CT after urethrogram shows the persistence of contrast in corpus spongiosum (arrow). **c** Sagittal T2-weighted MRI confirms edema and blood products within the corpus spongiosum (arrow)

Urethral injury can be seen in approximately 1–38% of penile fracture [19]. Penile fracture refers to rupture of corpus cavernosa with tunica albuginea tear, usually as a result of rapid blunt trauma to the erect penis. Clinically, the patient may present with penile swelling, pain and hematuria post intercourse. The morphology of the penis is described as an “eggplant penis” (Fig. 7). RUG is recommended in these cases, albeit it may be false negative in 28% of cases due to tamponade by hematoma [20]. Many urologists prefer direct visualization of urethra during penile fracture repair or urethroscopy [19]. Alternate option RUG shows urethral disruption with contrast extravasation into the corpus spongiosum. An urethrocavernous fistula may result with contrast opacification of the corpora cavernosa. CT after urethrogram may demonstrate persistence of contrast in corpus spongiosum, while MRI confirms edema and blood products within the corpus spongiosum (Fig. 8).

## Urethral inflammatory diseases

Gonococcal urethritis is a sexually transmitted disease caused by Neisseria gonorrhoeae. It commonly presents with purulent discharge. It often leads to complications like strictures which can be severe.

Non-gonococcal urethritis is most commonly caused by Chlamydia Trachomatis, and unlike gonococcal urethritis, is associated with only scanty non-purulent discharge. Complications are less severe as compared to gonococcal infections.

Chronic inflammatory urethritis may result in strictures (15%). Other reported complications include periurethral abscess, pseudodiverticulum, fasciitis, and Fournier gangrene. Urethro-perineal fistulas are more common with tuberculosis and schistosomiasis, although severe non-tuberculous infections may also result in fistulae.

**Fig. 9:**
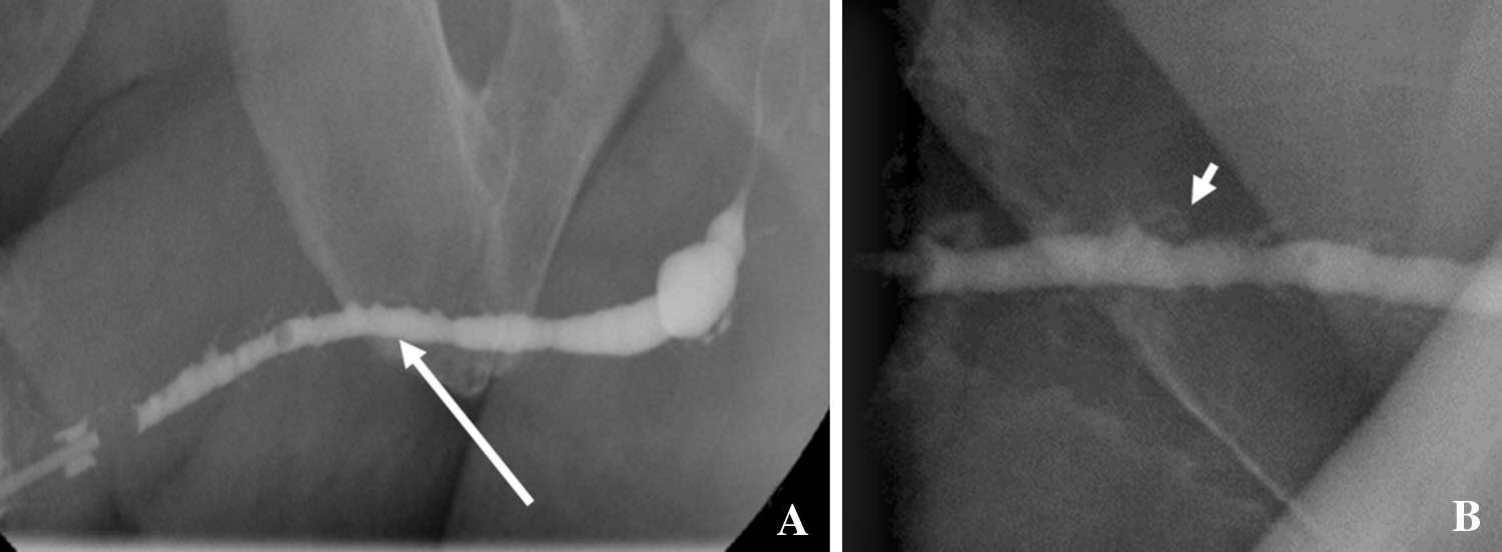
Retrograde urethrogram (**a**, **b**) in a case of urethritis demonstrates long segment irregular urethral narrowing (long arrow) associated with Littre gland dilatation (short arrow)

RUG in a case of urethritis typically demonstrates multifocal segmental or long segment irregular urethral narrowing. There may be opacification of the glands of Littre; visualization of the glands of Littre is quite specific for inflammatory urethritis. There may be retrograde opacification of the Cowper’s duct, which is usually related to high voiding pressures due to the obstruction caused by the strictures, with resultant reflux into the Cowper’s ducts [2, 3] (Fig. 9).

Other inflammatory conditions include condyloma acuminata, a viral infection causing venereal warts. Clinically, they present as urethral sessile polyps (papillomas) over the penis. Urethral involvement is seen in 0.5 to 5%. Theoretically any instrumentation including catheterization and RUG may lead to dissemination of infection to the proximal urinary tract and hence needs to be avoided during active infection. On imaging, small papillary filling defects in anterior urethra may be observed [21].

**Fig. 10:**
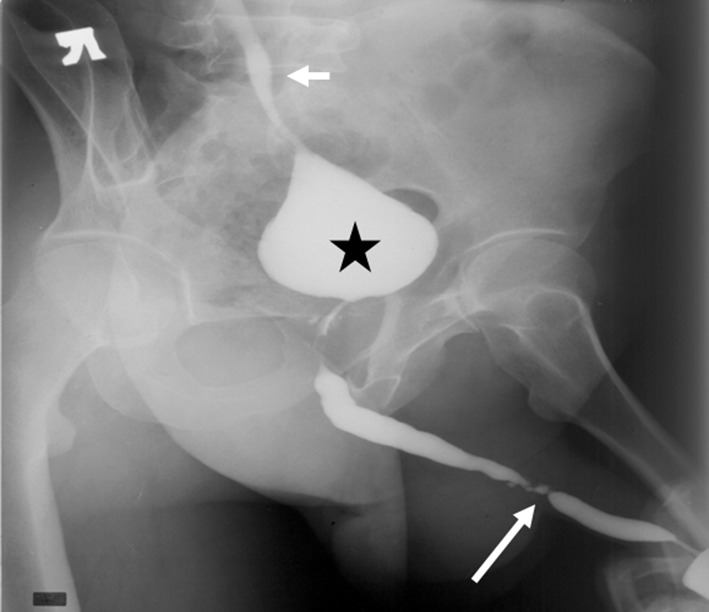
Retrograde urethrogram in an adult male with genitourinary tuberculosis demonstrates a thimble bladder (asterisk), ureteric strictures (long arrow), and vesico-ureteric reflux (short arrow)

Tuberculosis rarely involves the urethra and is usually a descending infection from the kidneys. The acute inflammatory phase is characterized by urethral discharge, and associated with prostatitis and epididymitis. The chronic stricturing stage presents with periurethral abscesses, perineal and scrotal fistulas, and a ‘watering can’ perineum. RUG may demonstrate prostato-cutaneous and urethro-cutaneous fistulas, in addition to a small-volume contracted bladder (“thimble bladder”), vesico-ureteric reflux, and ureteric strictures [22] (Fig. 10).

## Urethral strictures

Fibrous strictures of the urethra are pathologically characterized by spongial fibrosis, which refers to scarring of the periurethral corpus spongiosum [1]. Clinically, the patient may present with decreased urinary stream, hesitancy, and incomplete evacuation.

Anterior urethral strictures may be the result of inflammatory causes or result from trauma (straddle injury). Traumatic catheterization with resulting strictures is most common at the bulbo-membranous junction, followed by the penoscrotal junction. Congenital strictures are rare. If a long-term transurethral Foley catheter is not well supported and anchored to the thigh, pressure necrosis can occur, with resultant strictures frequently seen at the penoscrotal and bulbo-membranous junctions.

Posterior urethral strictures may be traumatic or iatrogenic (“bladder neck contracture”) after prostate surgery (TURP or prostatectomy) [21].

**Fig. 11:**
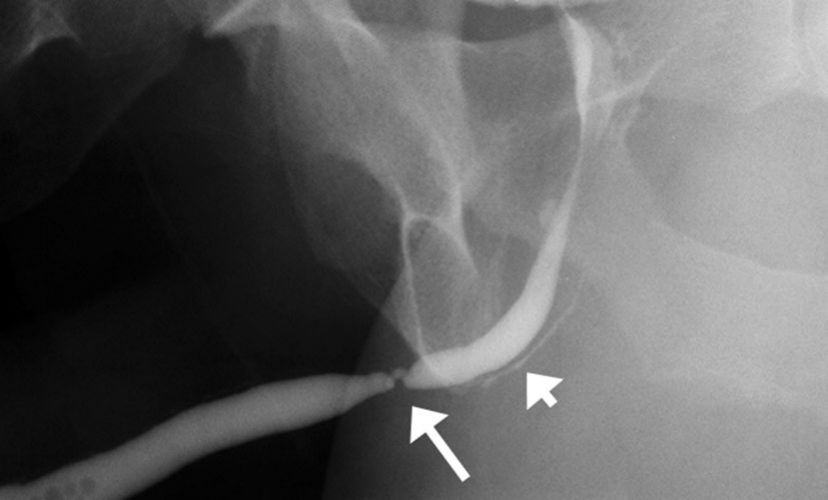
Retrograde urethrogram in a case of poor urinary stream. There is a high-grade stricture in the bulbar urethra (long arrow) with retrograde opacification of the Cowper’s duct (short arrow)

RUG helps in assessing the location, length, number, and degree of strictures, as well as periurethral abnormality (Fig. 11). The length of the stricture is a very important factor for determining management. Strictures that are 2 cm or less in length can initially be incised with endoscopic guidance—this is referred to as optical internal urethrotomy. Longer strictures often require open surgical repair, with mucosal grafts sometimes needed to bridge the strictured region. Sonourethrography and MR urethrography are helpful in accurate determination of the length of strictures and periurethral spongiosal fibrosis. However, they are not widely used to determine surgical management decisions in clinical practice. A combination of RUG and VCUG is usually necessary for assessing posterior urethral strictures [23]. MR imaging also accurately estimates the length of the prostatomembranous defect [10].

A combination of RUG and VCUG is usually necessary for assessing posterior urethral strictures, so that the length of involvement can be accurately assessed. MR imaging is also helpful in accurately estimating the length of the prostatomembranous urethral defect.

**Fig. 12:**
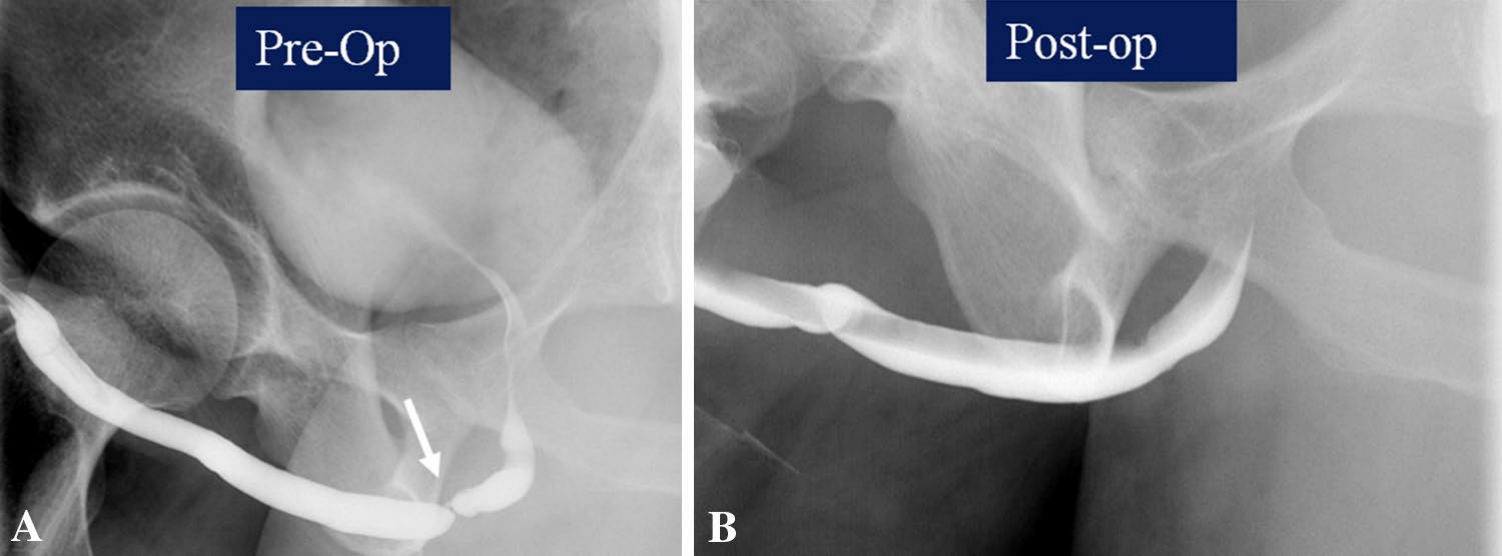
Pericatheter urethrogram. **a** Pre-operative retrograde urethrogram demonstrates short bulbar stricture (arrow). **b** Post-urethroplasty pericatheter study shows good opacification with no contrast extravasation

**Fig. 13:**
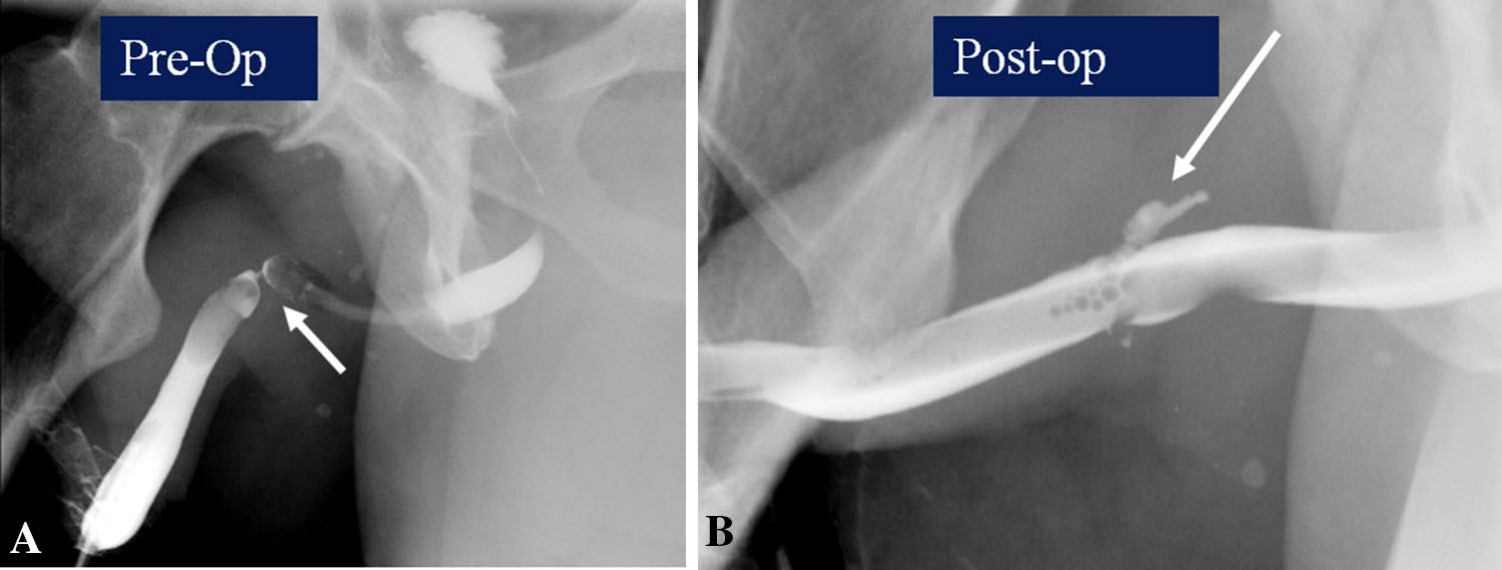
Pericatheter urethrogram. **a** Pre-operative retrograde urethrogram demonstrates tight bulbar stricture (arrow). **b** Post-urethroplasty pericatheter study shows good opacification with contrast extravasation (arrow)

Evaluation is often requested following surgical management of urethral strictures. A ‘pericatheter study’ is often performed in the post-operative situation, where a small caliber—6–8 French, soft feeding tube is inserted into the distal penile urethra alongside the indwelling Foley catheter, and contrast injected to assess for extravasation at the site of the stricture repair. If no leak is seen, at our institution, the bladder is then filled through the indwelling Foley, and the patient asked to void around the catheter to confirm absence of leakage at the repair site. The first post-operative study is also useful to assess the post-surgical luminal diameter (Figs. 12 and 13).

## Congenital urethral abnormalities

Various congenital abnormalities that can found in the urethra include prostatic utricle, posterior and anterior urethral valves, anterior urethral diverticulum, anorectal malformations with associated urethral fistula, meatal stenosis, hypospadiasis and epispadiasis and urethral duplication. Among these, congential urethral anomaly that can be seen in adults include prostatic utricle and mullerian duct cysts.

### Prostatic utricle

It is a common incidental congenital abnormality located at the verumontanum between the openings of the ejaculatory ducts. It communicates freely with the urethral lumen and can be associated with hypospadiasis and Eagle Barrett (prune belly syndrome). Transrectal ultrasound can detect prostatic utricle and MRI can be useful in detecting small ones and to demonstrate the urethral communication. A close differential is the Mullerian duct cyst which does not communicate with the urethra and often extends superior to the prostate. VCUG and RUG can depict the utricle and its size [24, 25].

## Urethral calculi

**Fig. 14:**
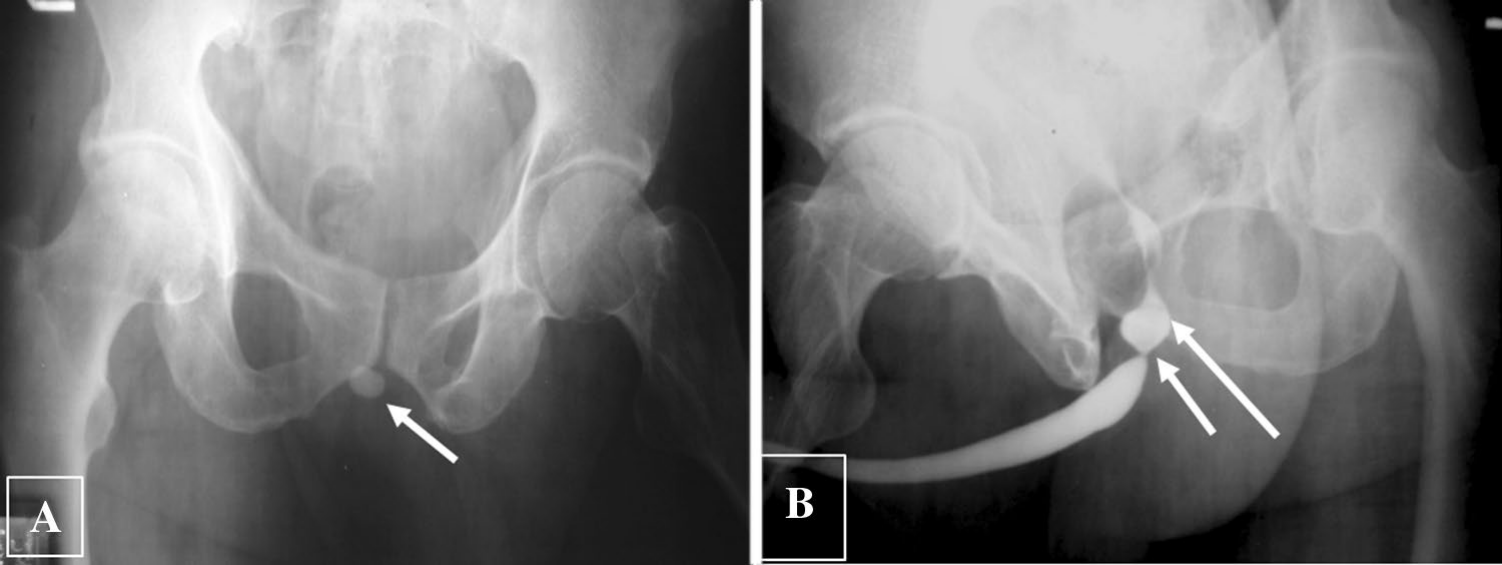
Urethral calculus. **a** Plain abdominal radiograph demonstrates a urethral calculus close to pubic symphysis (arrow). **b** Retrograde urethrogram depicts the urethral calculus as a rounded filling defect (long arrow) within the urethra proximal to a stricture (short arrow)

**Fig. 15:**
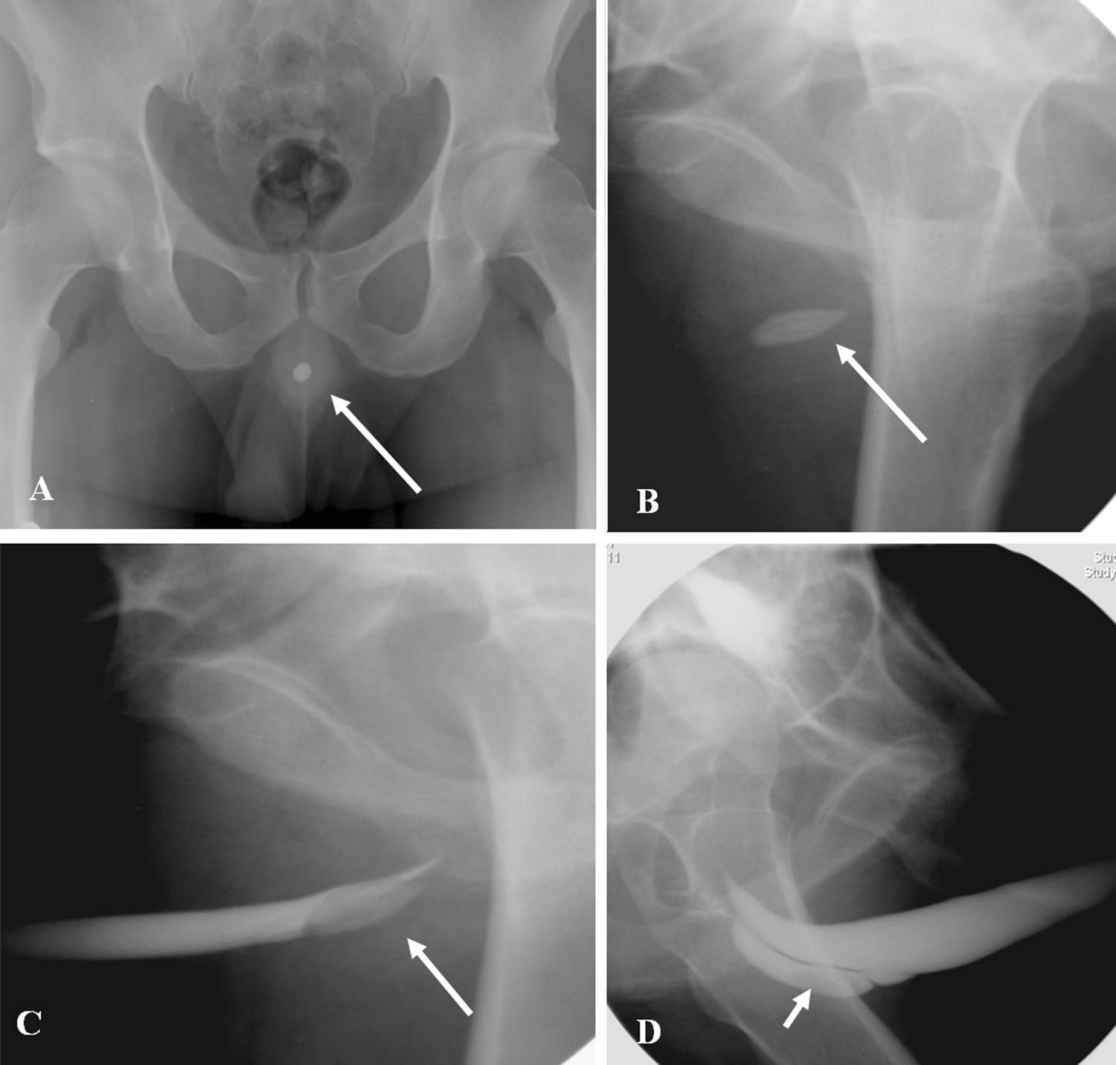
Calculus in Cowper’s duct syringocele. **a**, **b** Frontal and oblique plain radiograph shows radio opaque focus just below pubic symphysis along urethral course (arrows). **c**, **d** Retrograde urethrogram shows the calculus along the ventral aspect of proximal bulbar urethra (arrow) with extrinsic indentation. On further opacification (**d**), it is masked by the dense contrast in the dilated Cowper’s duct suggesting syringocele (short arrow)

These are usually calculi that have been expelled from the bladder (migrant calculi) and lodge at points of narrowing (such as the membranous urethra, external urethral meatus, or proximal to a urethral stricture). Rarely, urethral calculi may form in diverticula or proximal to a stricture (Figs. 14 and 15).

The radiological investigations include a good quality KUB radiograph which may demonstrate a calculus, and RUG may demonstrate a rounded or irregular filling defect within the urethra, sometimes proximal to a stricture, or within a periurethral diverticulum [2, 26].

## Complications of urethrography

Urethrography (RUG and VCUG) are not without complications. A temporary burning sensation and difficulty passing urine may be reported by the patient which usually resolves with time and reassurance. Infection (urethritis) as a complication is rare. Traumatic instrumentation with contrast extravasation has also been described specially in cases of difficult catheterization or underlying stricture. Venous intravasation due to urethral mucosal disruption by the inflated Foley balloon in the glans or the pressure of contrast injection especially in the presence of strictures may occur, can lead to opacification of the corpus spongiosum and draining pelvic veins. As with any contrast study, allergic reaction to contrast may occur due to systemic absorption [3, 21].

## Pitfalls of urethrography [1, 27, 28] (Figs. 16 and 17)

- Gas bubbles inserted through the catheter can mimic filling defects or polyps.
- Short pseudo strictures may result from urethral kinking due to inadequate penile traction or insufficient oblique position.
- False estimation of stricture length may result if RUG or VCUG are used in isolation. It is good practice to combine RUG and VCUG for assessing the length of strictures.
- External urethral sphincter contraction and bulbocavernous (constrictor nudae) spasm may mimic stricture. These are usually overcome by gentle steady pressure of injection.
- Opacification of the prostatic ducts, Cowper ducts, and periurethral glands of Littre may be mistaken for extravasation.
- High pressure injections may lead to contrast intravasation into the spongiosal plexus, and may mimic contrast leak.

**Fig. 16:**
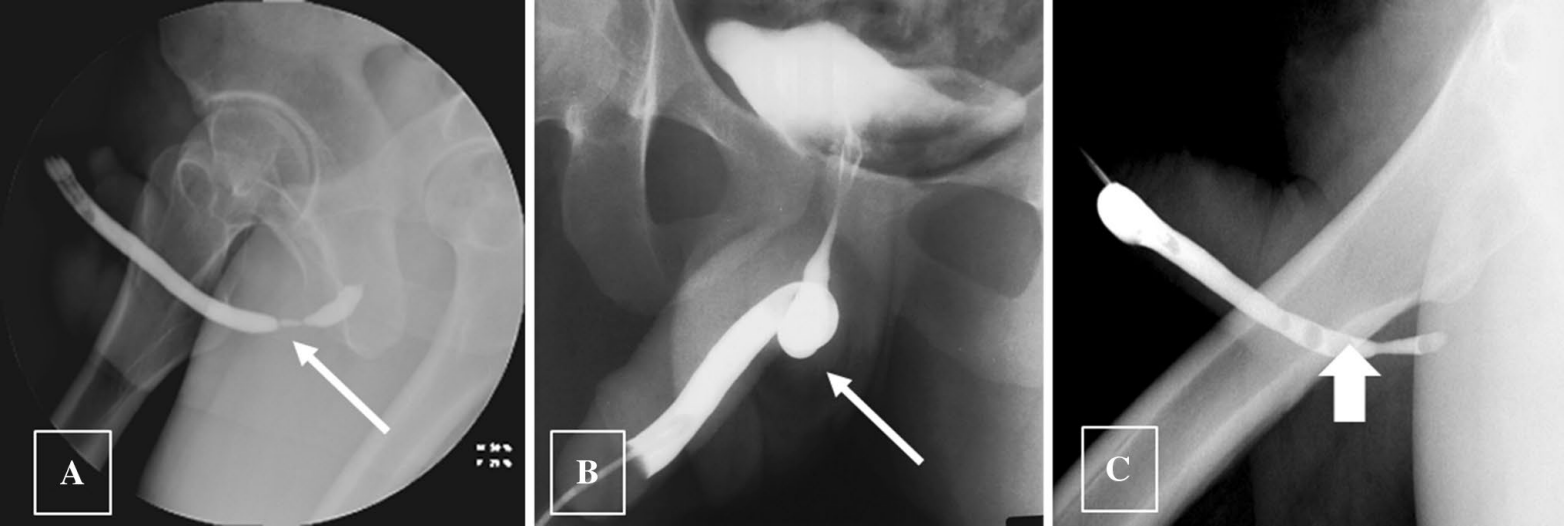
Pitfalls of retrograde urethrogram. **a** Bulbocavernous spasm (arrow) may mimic stricture, and is usually overcome by gentle and firm pressure of injection. **b** Pseudo stricture (arrow) may result from inadequate traction. **c** Air bubbles (thick arrow) injected into the urethra mimicking polyps

**Fig. 17:**
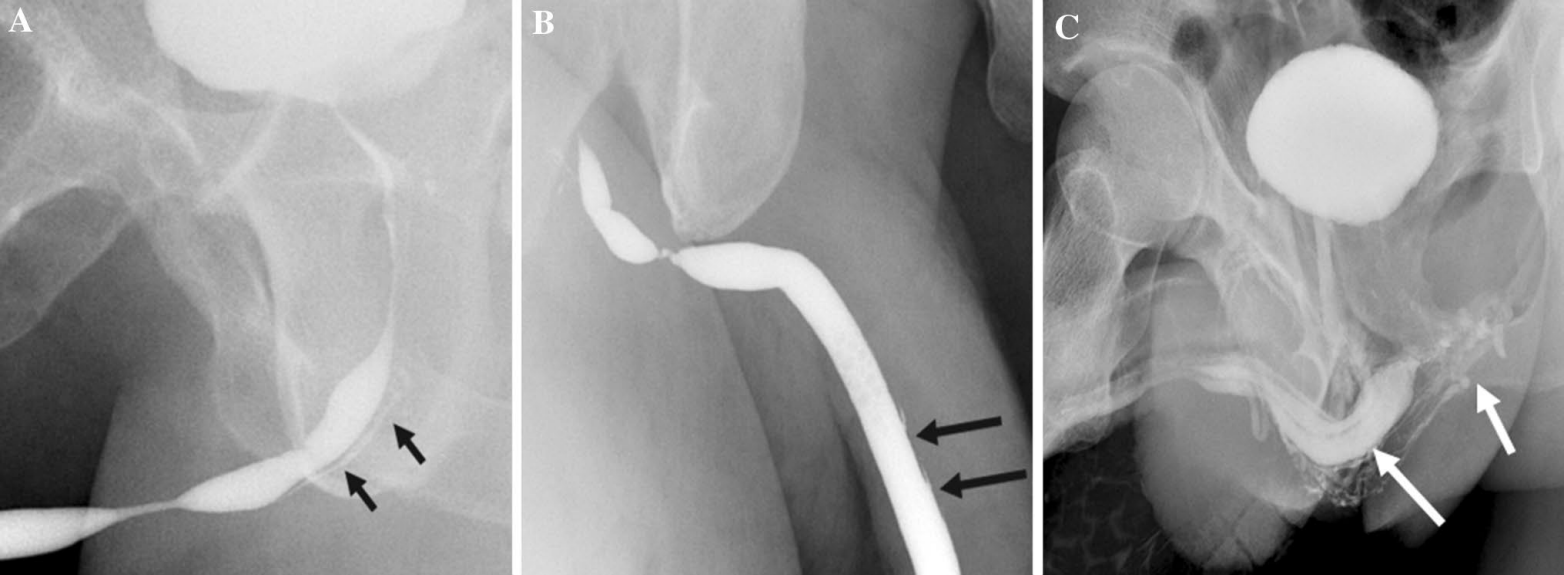
Normal structures in retrograde urethrogram mimicking urethral contrast extravasation. **a** Opacification of the Cowper ducts (arrow). **b** Periurethral glands of Littre (arrow). **c** High pressure injections may lead to contrast intravasation into the corpus spongiosa (long arrow) and venous plexus (short arrow)

## Penile prostheses

Penile prostheses (PP) are the third line therapy for erectile dysfunction after the failure of pharmacotherapy and vacuum devices. It gives high degree of patient satisfaction rate albeit an expensive and complex surgical procedure [29].

### Types of penile prostheses

There are two kinds of prostheses currently in use

1. Malleable PP (MPP) or semi-rigid PP is a simple non-inflatable paired malleable rods surgically placed in each of the corpora cavernosa. Advantages include easy surgical placement, less expensive, lower mechanical device failure and easy handling for the patients. Permanent erection due to rigid rod is the major drawback [30] (Fig. 18)

**Fig. 18:**
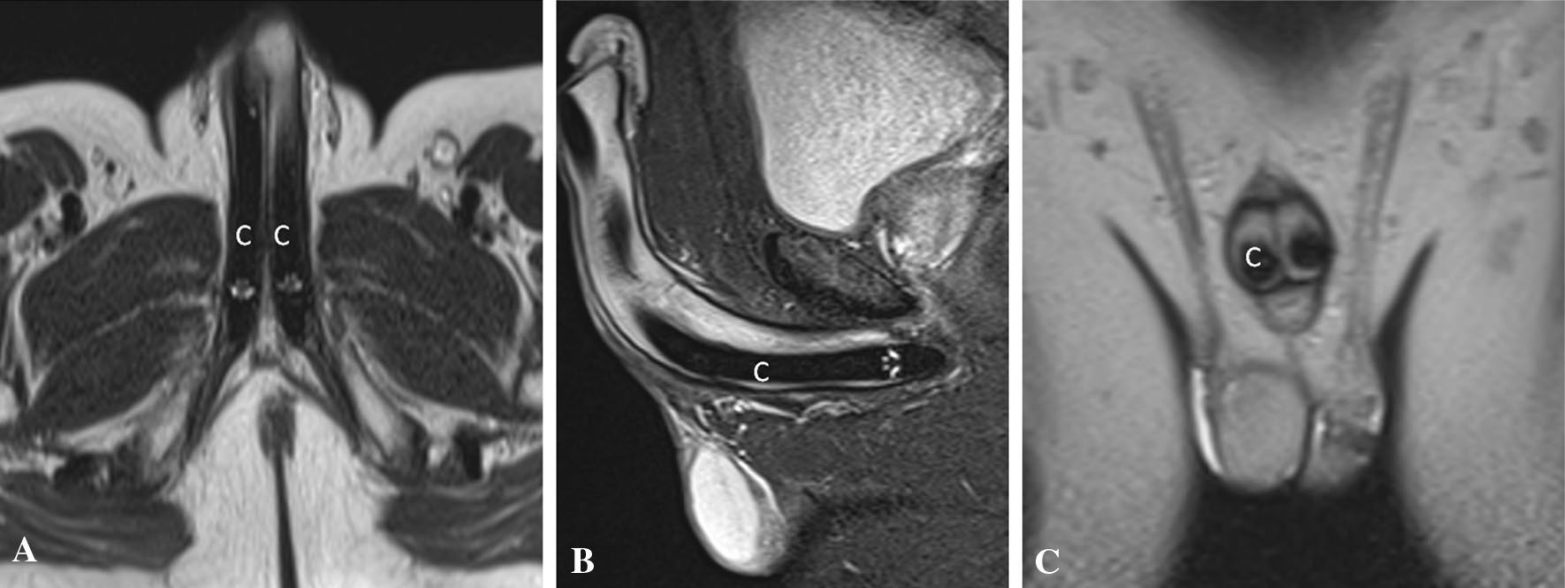
Appearance of normal malleable penile prosthesis in a 70-year-old male. Axial (**a**), sagittal (**b**), and coronal (**c**) T2-weighted MR images show paired T2 hypointense non-inflatable malleable penile prosthesis cylinders (**c**) in corpora cavernosa
2. Inflatable PP (IPP) can be of two types: (1) 3-pieces inflatable consisting of two inflatable cylinders placed inside corpora, a scrotal pump and a pelvic reservoir, (2) 2-piece inflatable containing two cylinders and a resipump (combined pump and reservoir) placed in the scrotum. All the components are interconnected by silicone tubing and are usually filled with normal saline [30] (Fig. 19).

**Fig. 19:**
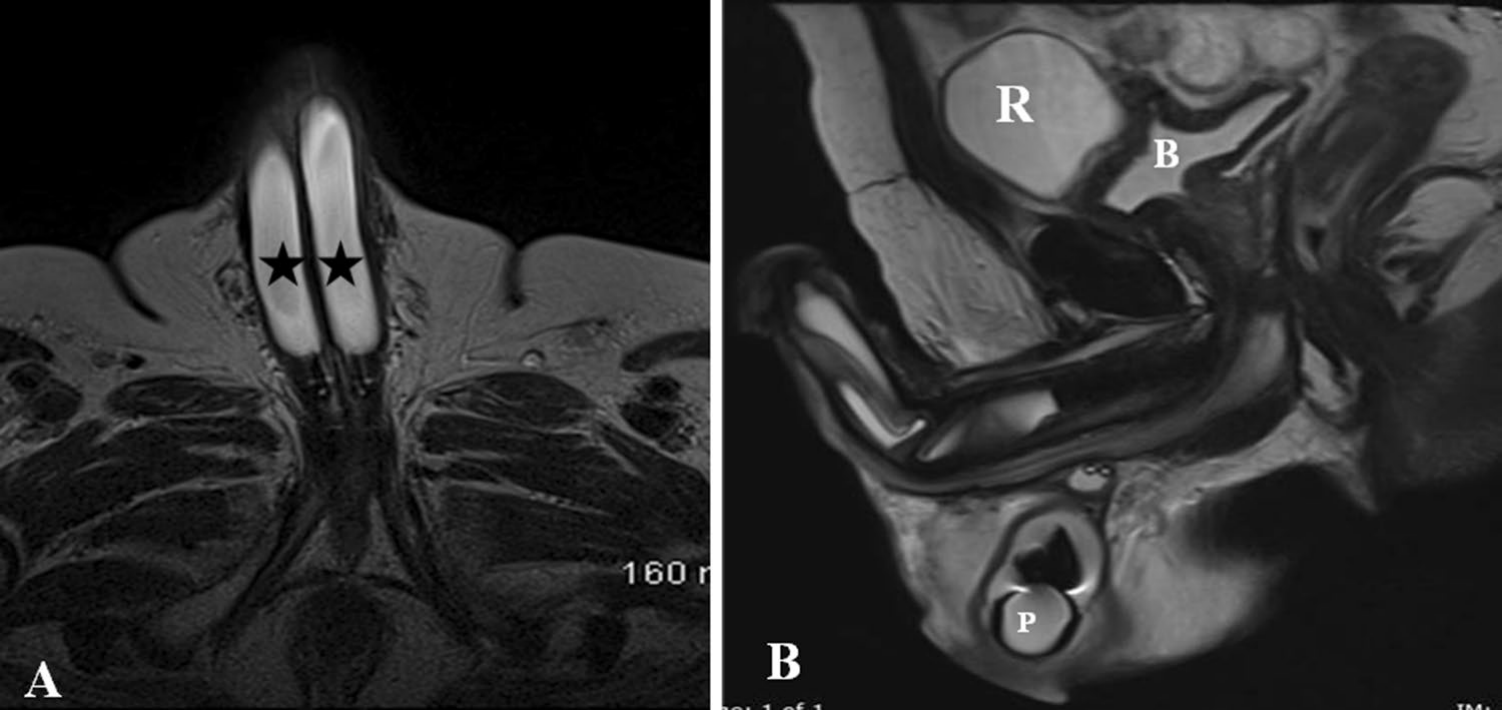
Appearance of normal inflatable penile prosthesis in a 65-year-old diabetic male. **a** Axial T2-weighted MRI shows the fluid filled corporal cylinders (asterisk). **b** Sagittal T2-weighted MRI shows the pelvic reservoir (R) adjacent to urinary bladder (B) and scrotal pump (P)

### Role of imaging

MRI is the modality of choice in the evaluation of penile prostheses. Fluid filled components in the IPP appear homogenously hyperintense on T2W images with T2 hypointense silicone covering. The proximal non-inflatable segment of the cylinders is rear tip extender and appears T2 hypointense. MPP appears predominantly hypointense on both T1- and T2-weighted images due to the metallic core [31, 32]. CT has limited role as it poorly depicts the internal architecture. It is useful in the evaluation of pelvic reservoir and in suspected infection in the implant. Ultrasound can be a useful technique in experienced hands and it can show the inflatable fluid filled penile cylinders, pelvic reservoir and scrotal pump. Radiography is obsolete now although it can show the fractures and malpositioning of malleable PI [30, 32].

Imaging is widely used in the evaluation of PP dysfunction to detect the complications. Rarely, it can be used in the assessment of degree of fibrosis in corpora cavernosa before implant placement.

Complications can be broadly categorized into three groups.

#### Device failure

This can be due to manufacture defect, misuse, or wear and tear. Fracture is a unique complication of MPP and can be diagnosed clinically as a palpable defect. CT and MRI can be useful in showing small defects. Similar complication in IPP is due to rupture of silicone covering of inflatable cylinders leading to leakage of saline. MRI can clearly show the collapsed or inadequate inflation of cylinders with kinking and adjacent fluid pocket. If some of the layers are intact, this leads to focal bulging of cylinders known as aneurysm. Similar complications can occur in pelvic reservoir as well. Most of these complications warrant implant removal and replacement [33].

#### Device malpositioning

**Fig. 20:**
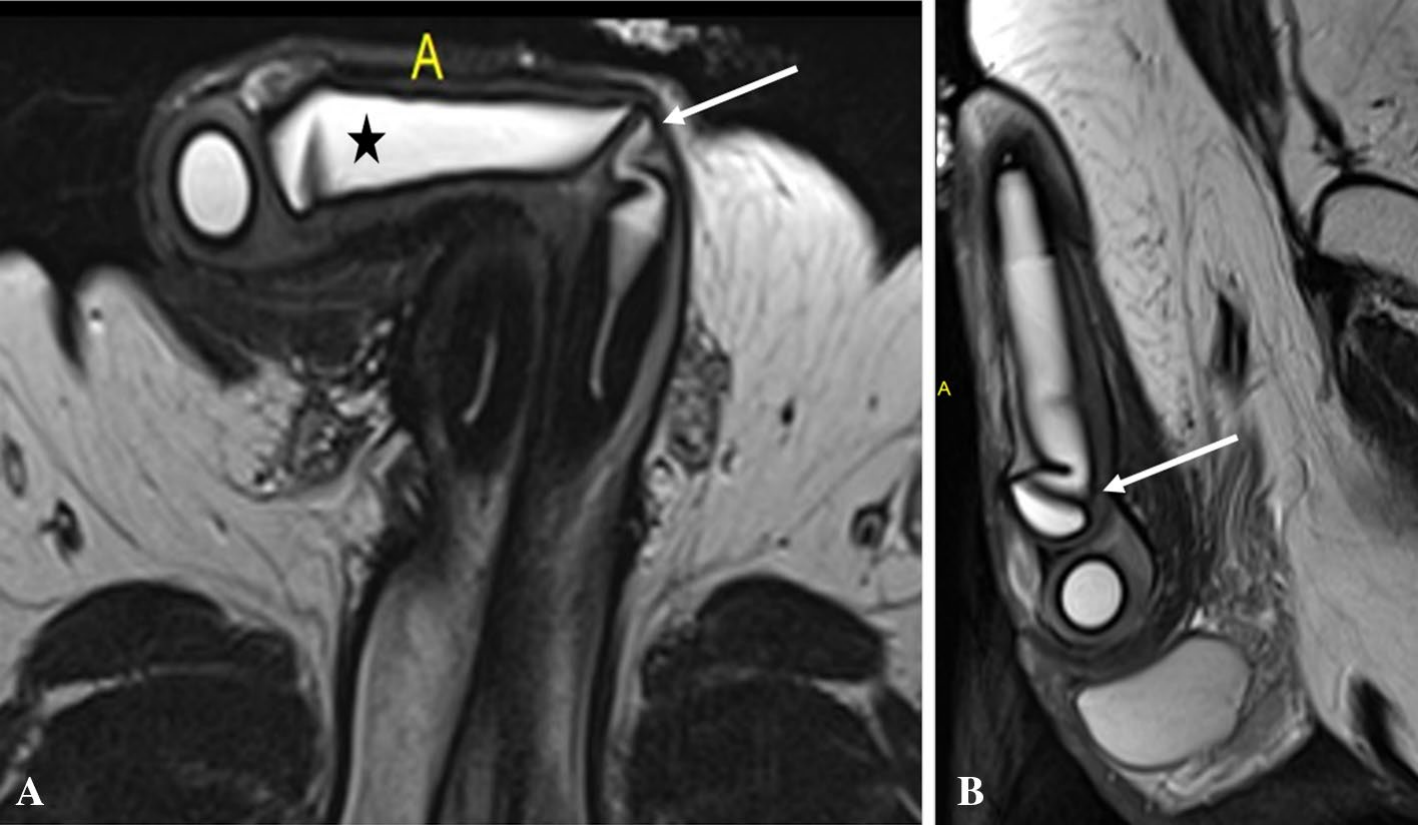
70-year-old man who had penile prosthesis insertion 6 years back presented with poor erection. Axial (**a**) and sagittal (**b**) T2-weighted MRI shows the buckling deformity (arrows) in the posterior part of one of the cylinders (asterisk)

**Fig. 21:**
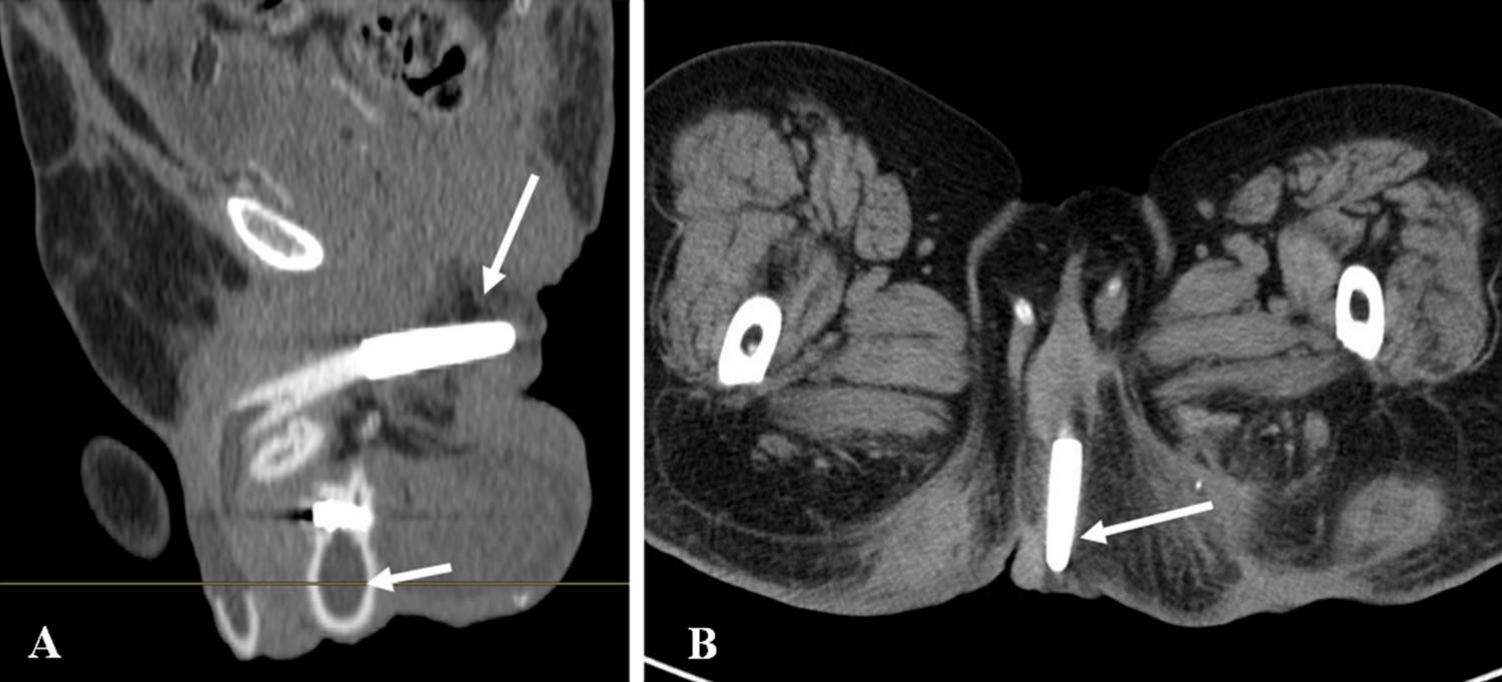
75-year-old man with penile prostheses inserted 10 years back after radical prostatectomy. Sagittal (**a**) and axial (**b**) CT images show the significant posterior migration of one of the cylinders (arrow) reaching the intergluteal cleft. Scrotal pump also seen (short arrow)

**Fig. 22:**
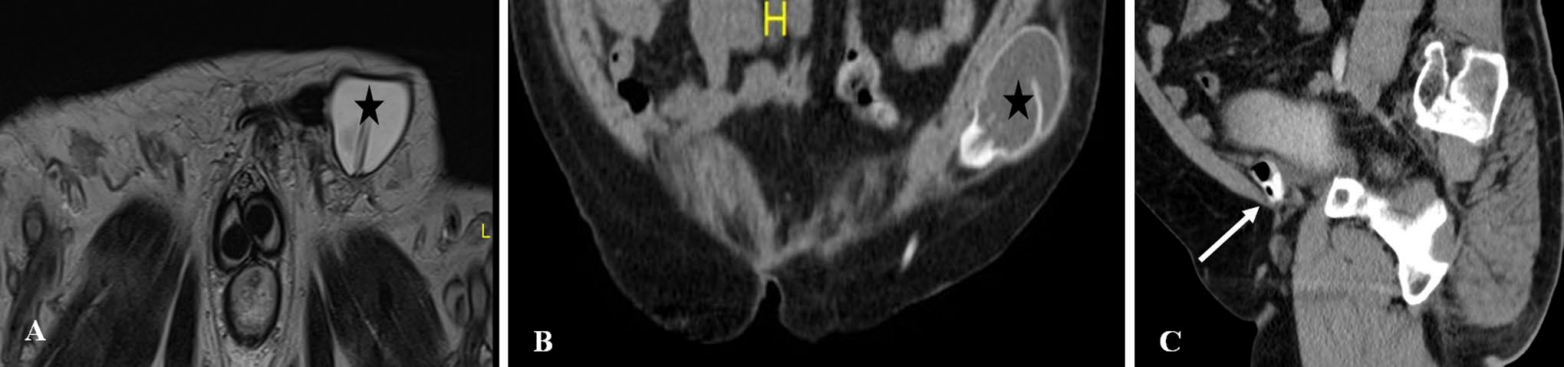
Complications involving reservoir. **a** Axial T2-weighted MRI shows the inguinal migration of pelvic reservoir (asterisk). **b** Coronal CT shows the abdominal wall placement of reservoir (asterisk) instead of usual pelvic placement. **c** Sagittal CT shows the collapsed contrast filled reservoir with air indicating reservoir failure (arrow)

This is a relatively common complication and can happen during intraoperative placement or later due to migration. Using inappropriate length of cylinders can lead to penile deformity in the form of S deformity with buckling in longer cylinders and floppy glans or Concorde deformity in shorter cylinders. Cylinders can migrate proximally, distally and laterally with or without erosions leading to deformity and poor erection (Figs. 20 and 21). One cylinder can cross over into the other corpora due to defect in the intercavernosal septum. These complications can be suspected clinically and often needs imaging for confirmation. One of the known complications of pelvic reservoir is migration into the inguinal canal or subcutaneous plane and can be mistaken for other pathologies [32, 34] (Fig. 22).

#### Infection

**Fig. 23:**
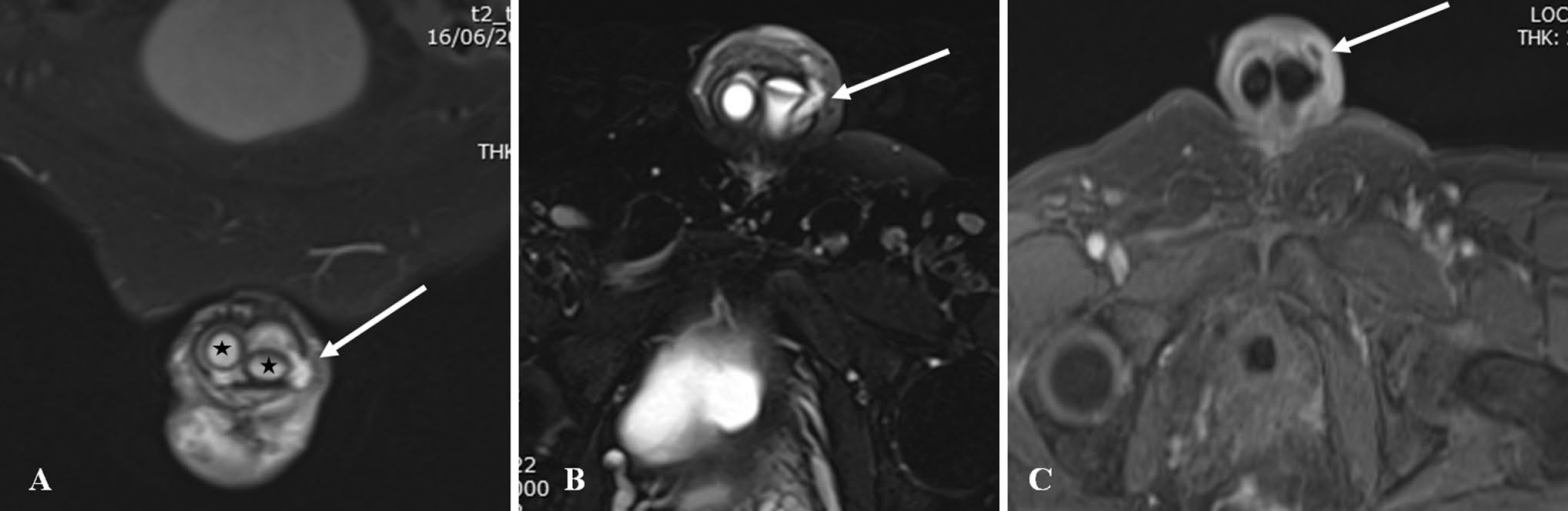
59-year-old diabetic man presented with wound discharge close to tubing site 25 days after penile prosthesis placement. Coronal (**a**), axial (**b**) T2-weighted and axial post-contrast (**c**) MRI images show small fluid collection with peripheral enhancement extending through the tunical defect into subcutaneous plane (arrow). Two corporal cylinders are seen (asterisk)

Infection is a serious complication as it often needs implant removal. Diabetes, spinal cord injury and prior surgery are the major risk factors. PP infection can be early (< 6 weeks) or delayed (> 6 weeks).Early infection can be diagnosed based on clinical and laboratory features. Delayed infection can present with variable symptoms and severity, commonest being penile pain on erection. Imaging helps in identifying low-grade infection as the cause of penile pain. Ultrasound is limited and can show sizable fluid collections. CT is the widely available modality and can show the collection, gas pockets and enhancement. MRI is used selectively for further assessment of the implant status and extent of spread of infection [34, 35] (Fig. 23).

## Incontinence therapy

Urinary incontinence is a relatively common symptom in the aging male population, with the prevalence of daily urinary incontinence in older men reported to range from 2 to 11% with increasing prevalence reported with increasing age [36]. Prostate surgery, including radical prostatectomy and transurethral resection of the prostate (TURP), is the most common cause of stress urinary incontinence [36].

When medical management does not provide sufficient relief of symptoms, surgical options are considered for treatment. These include artificial urinary sphincters (AUS), periurethral bulking agents, and perineal slings. Complications of artificial urinary sphincters can be the result of device component malpositioning, malfunctioning device components, and infection. An imaging exam may be ordered to trouble shoot a malfunctioning or symptomatic device. Neurologic disorders as well as prior pelvic trauma can also be a cause of stress urinary incontinence. Urgency incontinence is often related to detrusor muscle dysfunction and is associated with a strong urge to urinate; it is managed with surgically implanted sacral nerve stimulation [33].

### Artificial urinary sphincters (AUS)

**Fig. 24:**
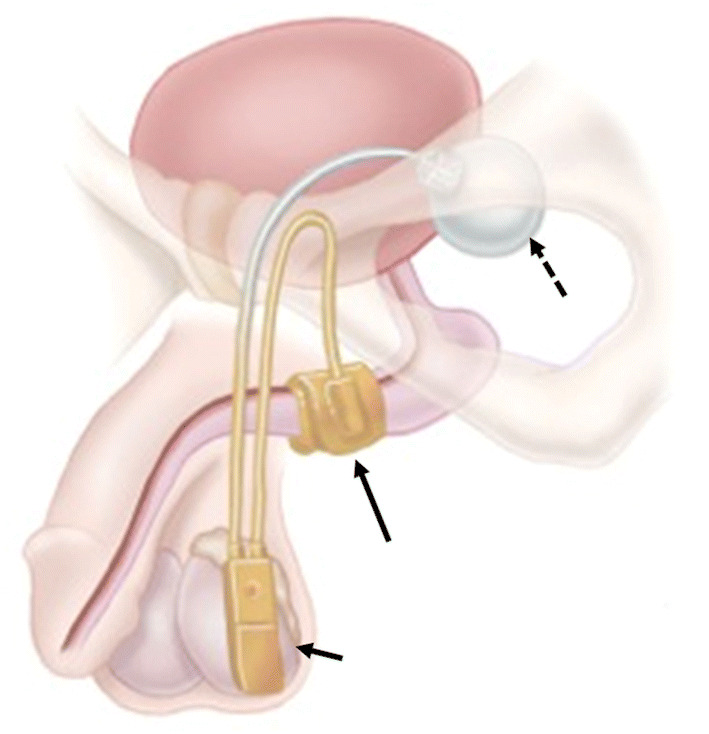
Photograph showing a normal artificial urinary sphincter (AUS) and its components namely inflatable cuff around the urethra (long arrow), reservoir in the pelvis (dashed arrow) and pump in the scrotum (short arrow). Reprinted, with permission, from Boston Scientific, Minnetonka, MN

**Fig. 25:**
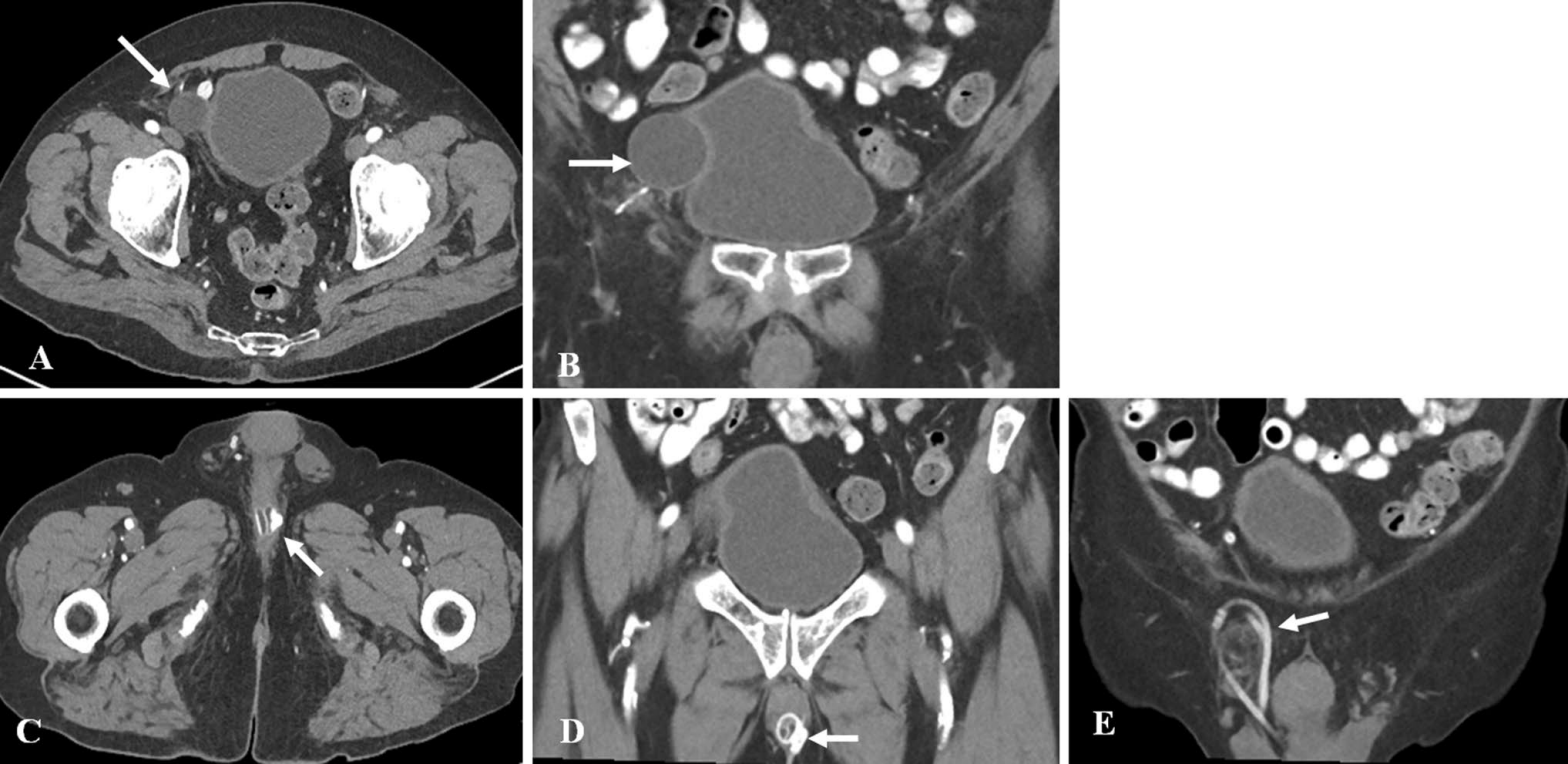
Appearance of uncomplicated AUS on CT in a 68-year-old male with florid incontinence after radical prostatectomy. **a** Axial contrast enhanced CT shows the reservoir (arrow) in the right pelvis in the space of Retzius. The reservoir indents the right side of the bladder, seen better on the coronal image (1b). It is important not to misdiagnose the reservoir as a cystic lesion in the pelvis. **b** Coronal view demonstrates the saline filled AUS reservoir (arrow) indenting the bladder. **c**, **d** Axial and coronal views more inferiorly shows the sphincter cuff (arrows) around the bulbar urethra. **e** Coronal view shows tubing related to the AUS coiled in right hemiscrotum (arrow)

The (AUS) consists of three main components: a control pump, Inflatable cuff, and a reservoir (which is also referred to as a pressure regulating balloon) (Fig. 24). In the past diluted contrast was used in the reservoir, but currently, the reservoir is filled with saline and will appear fluid attenuation on CT studies. Contrast filled AUSs were visible on pelvic radiographs and the reservoir and tubing were hyperattenuating on CT. The reservoir is preferentially placed in the space of Retzius (Fig. 25).

When the cuff is inflated, it occludes the urethra, and allows maintenance of continence—this is referred to as the activated position. To allow voiding, the AUS is deactivated by squeezing the scrotal pump which deflates the cuff and allows passage of urine through the urethra. After 90s, the cuff automatically re-inflates due to the existing pressure gradient between the reservoir and cuff [37].

*Periurethral bulking agents* can also be used to treat stress incontinence. Synthetic material, bovine collagen and autologous substances have all been used. The bulking agents are injected in the periurethral tissues to increase urethral cooptation by external pressure on the urethra. These agents are of limited efficacy in patients with severe incontinence. These agents may be identified in the periurethral region on MRI and rarely on CT [38].

*Slings* are typically made of synthetic silicone mesh or porcine dermal collagen and are placed in the suburethral region to suspend and kink the urethra. The slings can have two types of attachment: (1) the sling scar into place post-operatively and are not surgically attached to any structure, (2) the slings can be anchored to the pubic rami by bone anchoring sutures. The sling itself is not radiopaque and therefore not visualized on imaging. However, the bone anchors can be identified as anchor attachments to the inferior pubic rami. [37].

### Complications

Imaging is useful in assessing complications related to the AUS, and relatively unhelpful in assessing periurethral bulking agents. The reservoir may be malpositioned, and instead of being located within the preferred location of the space of Retzius, it may migrate into adjacent pelvic structures such as the urinary bladder and the inguinal canal. The position of the reservoir is best assessed by cross-sectional imaging [33].

The tubing of the AUS may become kinked or disconnected. When contrast was used to distend the reservoir of the AUS, the components of the AUS could be identified on radiographs and the discontinuity or kinking of tubing could be identified. With the current trend for saline distension of the reservoir, imaging is not helpful in assessing the cause of device malfunction [37].

**Fig. 26:**
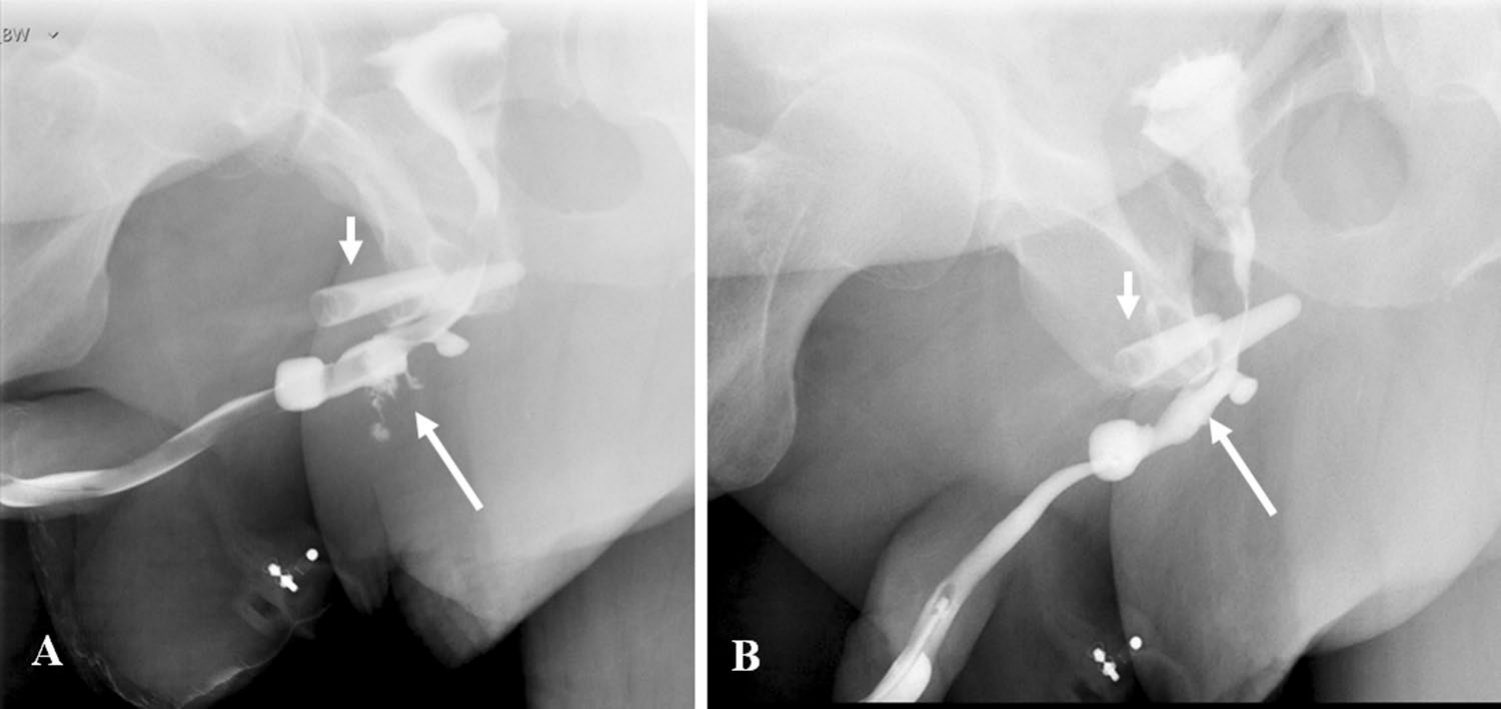
VCUG demonstrating extravasation from urethra at cuff site following explantation of an infected AUS. A 72-year-old man presented with scrotal erythema and tenderness a few months after AUS placement. Infection was suspected clinically and the AUS was removed in its entirety except for the pump mechanism in the scrotum. **a** VCUG shows extravasation from the urethra at the site of cuff explant (long arrow). The radiopaque crural struts of a penile prosthesis are also present (short arrow). **b** VCUG 2 months later shows healing of the extravasation. The post-operative urethral luminal irregularity will be a persistent finding (long arrow)

**Fig. 27:**
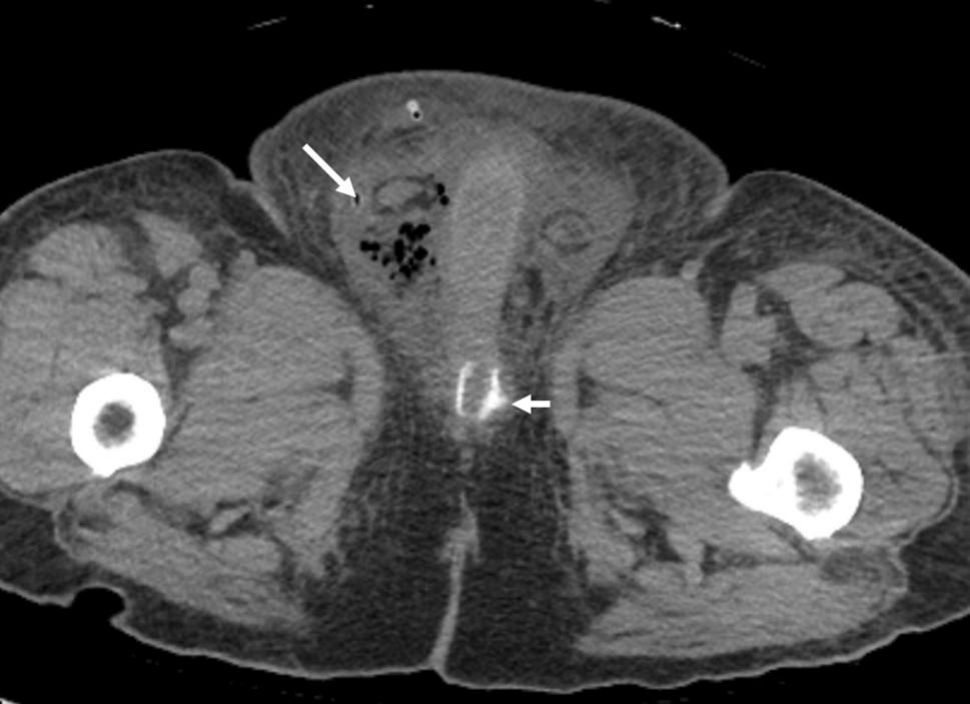
A 64 year-old presented with scrotal pain, redness and swelling. Axial CT shows the cuff around the bulbar urethra (short arrow). Multiple foci of gas in right hemiscrotum represented gas in an abscess (long arrow). The AUS was removed in its entirety and a Foley catheter placed for bladder drainage. VCUG done 2 months post-op (not shown) demonstrated complete urethral healing

**Fig. 28:**
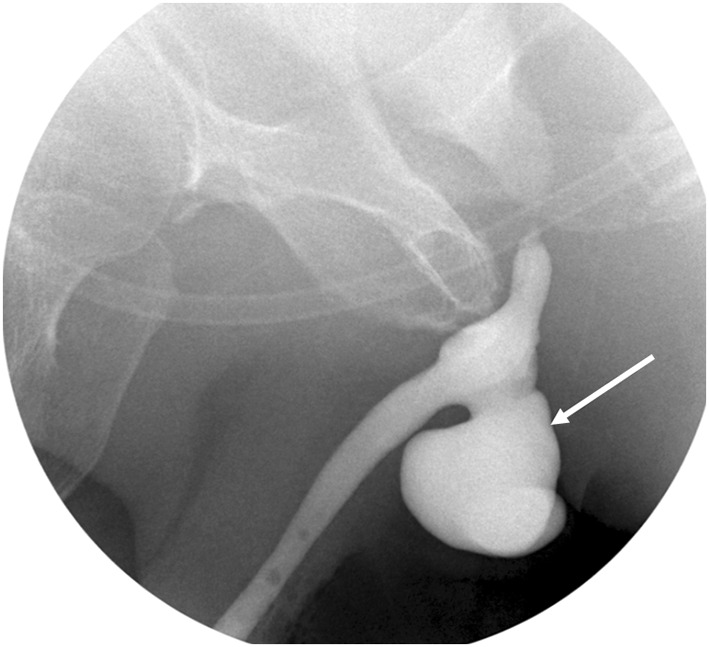
Fluoroscopic retrograde urethrogram shows a large diverticulum at the site of AUS cuff removal (arrow). Patient had two attempts at placement of an AUS, with removal necessary both times for infection

The sphincter cuff may erode into the urethra, usually due to infection in the operative bed. Patients present with pain and erythema in the perineum, and there may be frank abscess formation. Retrograde urethrogram demonstrates communication of the cuff with the urethral lumen, referred to as cuff erosion (Figs. 26 and 27). This complication necessitates removal of the entire AUS, with replacement once the infection is completely resolved. Urethral diverticula may develop at the site of cuff erosion [38] (Fig. 28).

## Conclusion

In spite of widespread usage of cross-sectional modalities in genitourinary imaging, urethrography still holds an important place. It is the imaging modality of choice in suspected urethral trauma and urethral strictures and also useful in surgical planning and evaluating post-operative complications. Radiologists must be aware of the anatomy, imaging appearances of various urethral pathologies, and pitfalls in image interpretation. A clinically oriented approach to imaging and reporting is helpful for the treating urologist.
